# Polymorphisms in the Regulatory Region of the Cyclophilin A Gene Influence the Susceptibility for HIV-1 Infection

**DOI:** 10.1371/journal.pone.0003975

**Published:** 2008-12-18

**Authors:** Maarten A. N. Rits, Karel A. van Dort, Neeltje A. Kootstra

**Affiliations:** Department of Experimental Immunology, Sanquin Research, Landsteiner Laboratory, and Center for Infectious Diseases and Immunity Amsterdam (CINIMA), Academic Medical Center, University of Amsterdam, Amsterdam, the Netherlands; National Cancer Institute, United States of America

## Abstract

**Background:**

Previous studies have demonstrated an association between polymorphisms in the regulatory regions of Cyclophilin A (CypA) and susceptibility to both HIV-1 infection and disease progression. Here we studied whether these polymorphisms are associated with susceptibility to HIV-1 infection and disease progression in the Amsterdam Cohort on HIV-1 infection and AIDS (ACS) in a group of men having sex with men (MSM) and drug users (DU).

**Methodology/Principal Findings:**

We screened participants of the ACS for the C1604G and A1650G polymorphisms in the regulatory regions of CypA. The prevalence of the 1650G allele was significantly higher in high risk seronegative MSM than in HIV-1 infected MSM. However, C1604G or A1650G were not associated with the clinical course of infection in MSM of the ACS. Interestingly, participants of the ACS-DU who carried the 1604G allele showed a significantly accelerated progression when viral RNA load above 10^4.5^ copies per ml plasma was used as an endpoint in survival analysis.

**Conclusion/Significance:**

The results obtained in this study suggest that the A1650G polymorphism in the regulatory region of the CypA gene may be associated with protection from HIV-1 infection, while the 1604G allele may have a weak association with the clinical course of infection in DU.

## Introduction

The peptidyl prolyl isomerase Cyclophilin A (CypA) is a cytoplasmic factor that is essential for efficient infection of HIV-1 [1]–[3]. It is specifically incorporated into the HIV-1 virion which is mediated through an interaction with the capsid protein of which an exposed loop between helices 4 and 5 mimic a natural ligand for CypA [4], [5]. Although CypA is incorporated in the virion, the presence of CypA in the target cell has the more significant effect on virus replication [6]–[9]. The molecular mechanism by which CypA enhances infection is largely unknown. CypA can catalyze the cis/trans isomerization of prolyl-peptide bonds in the HIV-1 capsid protein [10], which suggests that CypA has a possible role in uncoating of the viral core following entry into the cytoplasm.

Recently, CypA has gained a lot of interest when it was identified as a cofactor for the tri-partite containing motif (Trim)5α in several simian species [11]–[14]. Trim5α is a retroviral restriction factor that associates with the capsid protein and blocks HIV-1 infection at an early step following entry of the viral core into the cytoplasm [15]. Moreover, Trim5-CypA fusion proteins have been discovered in several simian species [16], [17], suggesting that CypA in some instances can function as a capsid recognition domain for antiretroviral proteins.

Polymorphisms in human genes that serve as HIV-1 cofactors or restriction factors have been described to influence susceptibility to HIV-1 and disease progression. For example, polymorphisms in chemokine receptors that serve as HIV-1 coreceptors and their natural ligands have been associated with susceptibility to infection as well as disease progression [18]–[21]. Recently, polymorphisms in cellular factors like Apobec3G, CUL5 and Trim5α, that are directly or indirectly involved in innate immunity have also been demonstrated to have an effect on the clinical course of infection [22]–[24].

Previously 11 polymorphisms in the CypA gene have been identified none of which were located in the coding region of the gene [25]. Two of these SNPs (A1604G and C1650G) might affect CypA expression levels based on their location in the promoter region of the CypA gene and these polymorphism have been demonstrated to affect susceptibility to HIV-1 infection and disease progression [25], [26]. Here we studied the role of SNPs in the regulatory region of CypA gene on HIV-1 susceptibility and course of HIV-1 infection in participants of the Amsterdam Cohort Studies on HIV-1 infection and AIDS (ACS).

## Results

### Distribution of the regulatory polymorphisms C1604G and A1650G in the CypA gene and the effect on susceptibility to HIV-1 infection

The haplotype frequency of polymorphisms C1604G and A1650G in the regulatory region of the CypA gene was studied in three groups: HIV-1 positive MSM of the ACS (n = 334), MSM of the ACS who remained seronegative despite reported high-risk behavior (High-risk seronegatives, HRSN; n = 68), and HIV-1 negative blood donors (controls) (n = 104). For all groups, genotype distributions and minor allele frequencies are shown in Table 1. Although a higher allele frequency of C1604G was observed in the HIV-1 infected MSM as compared to the HRSN participants and controls, this difference was not statistically significant indicating that the C1604G is not associated with susceptibility to HIV-1 infection (Table 1).

**Table 1:** Genotype distributions of the CypA SNPs C1604G and A1650G and risk for HIV-1 infection.

SNP	Risk group	Number	Genotype frequency (%)			Controls or HRSN vs. HIV-1+MAJ vs. HZ+MIN	
			MAJ	HZ	MIN	OR (95% CI)	p-value
**C1604G**	HIV-1+	334	279 (83.3)	52 (15.5)	4 (1.2)		
	HRSN	68	60 (88.2)	8 (11.8)	0 (0.0)	0.66 (0.30–1.46)	0.31
	Controls	104	92 (88.5)	11 (10.6)	1 (1.0)	0.65 (0.33–1.26)	0.20
**A1650G**	HIV-1+	334	249 (74.6)	75 (22.5)	10 (3.0)		
	HRSN	68	42 (61.8)	21 (30.9)	5 (7.4)	1.82 (1.05–3.14)	0.03
	Controls	104	74 (71.2)	23 (22.1)	7 (6.7)	1.19 (0.73–1.94)	0.48

MAJ = major genotype; HZ = heterozygous genotype; MIN = minor genotype.

The minor allele frequency of the A1650G polymorphism in the HRSN was significantly increased as compared to HIV-1 positive MSM, suggesting that the 1650G allele may be associated with a decreased susceptibility to HIV-1 infection in participants of the ACS.

### Effects of regulatory CypA polymorphisms on disease progression

Next, we examined the influence of polymorphisms C1604G and A1650G on the clinical course of infection in HIV-1 positive MSM of the ACS. Kaplan Meier and Cox proportional hazard analyses were used to test for potential differences in rates of progression to CD4+ T cells below 200 cells per µl, clinical AIDS according to the CDC definition of 1987 and 1993 [27], [28] or plasma viral RNA load above 10^4.5^ copies per ml between individuals who carried the major or minor allele (dominant model). The C1604G and the A1650G polymorphisms were not associated with survival time among MSM of the ACS, independent of the endpoints used for analysis (Table 2). In addition, both polymorphisms were also not associated with CD4+ T cell number or plasma viral RNA load at setpoint in HIV-1 positive MSM (data not shown).

**Table 2:** Effects of CypA SNPs C1604G and A1650G on disease progression in the Amsterdam cohort of MSM.

SNP	Endpoint	Number	Events	RH (95% C.I.)	p-value
**C1604G**	AIDS (CDC1987)	334	211	0.91 (0.63–1.31)	0.61
	AIDS (CDC1993)	333	235	0.84 (0.59–1.20)	0.34
	CD4<200 cells/µl	333	199	0.84 (0.57–1.23)	0.37
	RNA>10^4.5^ copies/ml	331	232	1.02 (0.71–1.45)	0.92
**A1650G**	AIDS (CDC 1987)	334	211	0.97 (0.71–1.33)	0.87
	AIDS (CDC1993)	333	235	1.07 (0.80–1.43)	0.67
	CD4<200 cells/µl	333	199	0.98 (0.70–1.36)	0.89
	RNA>10^4.5^ copies/ml	331	232	0.88 (0.65–1.20)	0.42

P-values were from Cox proportional hazard analysis.

Similar analyses were performed in the ACS of drug users (DU, n = 120). In this cohort, 9% of the individuals were heterozygous for C1604G (minor allele frequency of 0.046) and 25.4% were heterozygous for A1650G (minor allele frequency of 0.129). None of the individuals was homozygous for either 1604G or 1650G. The minor allele frequency for the C1604G and A1650G polymorphism among DU was similar to the frequencies observed in the controls (Table 1).

**Figure 1:**
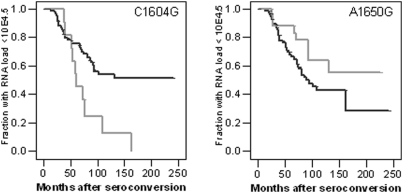
Survival analysis for C1604G and A1650G in DU of the ACS. Kaplan Meier analysis for time in months from seroconversion to viral RNA load above 10^4.5^ copies per ml plasma in DU of the ACS based on the C1604G genotype (left panel) or A1650G genotype (right panel). Black lines indicate individuals homozygous for the major allele (left panel: 1604CC; right panel: 1650AA); Gray lines indicate individuals heterozygous for the minor allele (left panel: 1604CG; right panel: 1650AG).

Also in participants of the ACS-DU, the minor alleles of C1604G and A1650G were not associated with progression to a first CD4+ T cell count below 200 cells per µl blood, or clinical AIDS (table 3). However, carriers of the minor allele of C1604G showed a significantly accelerated progression towards a viral RNA load above 10^4.5^ copies per ml plasma (log rank p = 0.007, RH 2.55, CI95% 1.252–5.171; p = 0.01) (Figure 1, table 3). DU carrying the A1650G minor allele tended to have a somewhat slower progression towards viral RNA load above 10^4.5^ copies per ml plasma, albeit not statistically significant (Figure 1, table 3). No significant association between CD4+ T-cells or RNA setpoint and the C1604G or A1650G polymorphism was observed (data not shown).

**Table 3:** Effects of CypA SNPs C1604G and A1650G on disease progression in DU of the Amsterdam cohort.

SNP	Endpoint	Number	Events	RH (95%CI )	p-value
**C1604G**	AIDS (CDC 1987)	120	41	1.32 (0.52–3.39)	0.56
	AIDS (CDC1993)	120	72	1.27 (0.61–2.65)	0.53
	CD4<200 cells/µl	120	54	1.42 (0.64–3.15)	0.39
	RNA>10^4.5^ copies/ml	120	46	2.55 (1.25–5.17)	0.01
**A1650G**	AIDS (CDC 1987)	120	41	1.31 (0.69–2.51)	0.41
	AIDS (CDC1993)	120	72	1.24 (0.75–2.06)	0.39
	CD4<200 cells/µl	120	54	1.27 (0.71–2.26	0.42
	RNA>10^4.5^ copies/ml	120	46	0.57 (0.27–1.18)	0.13

P-values were from Cox proportional hazard analysis.

### Effect of regulatory polymorphisms in CypA on CypA expression levels and HIV-1 replication

**Figure 2:**
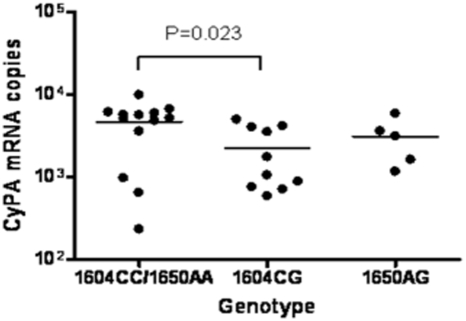
Analysis of CypA expression levels. CypA mRNA levels in PBMC obtained from healthy controls that were homozygous for the 1604 and 1650 major alleles (1604CC/1650AA), heterozygous for the 1604 minor allele (1604CG), or heterozygous for the 1650 minor allele (1650AG). CypA mRNA levels were normalized for β-actin mRNA levels.

Next we analyzed whether the C1604G and A1650G polymorphisms in the regulatory regions of the CypA gene were associated with altered CypA expression levels. To this end, we performed quantitative RT-PCR on CypA mRNA levels in peripheral blood mononuclear cells (PBMC) of 28 healthy blood donors genotyped for the C1604G and A1650G polymorphism. We observed a reduced CypA expression in PBMC that were heterozygous for the 1604G minor allele as compared to PBMC that were homozygous for the major allele (1604C) (Unpaired T-test; P = 0.023) (figure 2). No differences in CypA expression were observed in PBMC heterozygous for the 1650G minor allele compared to PBMC homozygous for the 1650A major allele.

**Figure 3:**
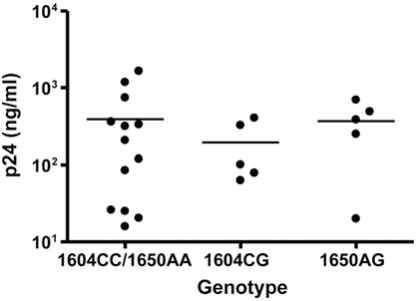
Analysis of HIV-1 replication in genotyped PBMC. Replication of NL4-3 Ba-L in PHA stimulated PBMC from healthy donors homozygous for the 1604 and 1650 major allele (1604CC/1650AA), heterozygous for the 1604 minor allele (1604CG), or heterozygous for the 1650 minor allele (1650AG). Virus replication was analyzed by measuring p24 production in culture supernatant. P24 production at day 12 after infection is shown.

Next, we analyzed the replicative capacity of HIV-1 in PHA-stimulated PBMC from healthy individuals with known genotypes for the C1604G and A1650G polymorphism. PHA-stimulated PBMC were inoculated with NL4-3 Ba-L (2×10^3^ TCID_50_ per 5×10^6^ cells). Subsequently, virus replication was assessed by measuring p24 antigen levels in the culture supernatant every other day for a period of two weeks. No significant differences in p24 production were observed at any time point during the culture period, irrespective of the genotype of the regulatory region of the CypA gene (figure 3).

## Discussion

CypA has been identified as a cofactor for HIV-1, leading to enhanced infection by a largely unknown mechanism [2]. The identification of Trim5-CypA fusion proteins as restriction factors [16], [17] and the dependency of Rhesus Trim5α on CypA has renewed the interest in this ubiquitously expressed cellular protein [11]–[14]. Here we analyzed the effects of polymorphisms in the promoter region of the CypA gene on susceptibility for HIV-1 infection and disease progression in participants of the ACS.

In a previous study, 1604G was observed to be associated with a more rapid CD4+ T-cell decline in African Americans and a trend towards more rapid progression to AIDS was observed in European Americans [25]. In our cohort of HIV-1 positive Caucasian MSM, no association between 1604G and progression to AIDS or any other endpoint was observed. This is in agreement with observations in the Swiss cohort of HIV-1 infected individuals who are also all Caucasian [26].

In the ACS on HIV-1 infected drug users, we could also not demonstrate an association between polymorphisms in the regulatory region of the CypA gene and survival to CD4+ T-cell counts below 200 cells per µl or AIDS according to the 1987 and 1993 definitions. In this cohort, however, time to plasma viral RNA load above 10^4.5^ copies per ml was significantly reduced in 1604G carriers. These observations suggest only a minor effect of polymorphisms in the regulatory region of CypA which may become apparent only during the late stages of infection when viral load is increasing.

A second polymorphism in the regulatory region of the CypA promoter involves position 1650. In a Swiss Caucasian cohort, 1650G was reported to be associated with a rapid CD4+ T cell loss and enhanced progression to disease [26]. An association between the presence of 1650G and delayed disease progression was also observed in a cohort of HIV-1 infected African Americans and a trend towards a delayed progression to AIDS was observed in European Americans [25]. We were unable to confirm these results in our cohort of MSM. However, we did observe an enrichment of the 1650G allele in MSM who have remained seronegative despite high risk behavior (HRSN) as compared to HIV-1 infected MSM which might indicate that the 1650G allele is associated with a reduced susceptibility to HIV-1 infection. This contradicts findings of an earlier study where a reduced prevalence of the 1650G allele was observed in European Americans who remained seronegative despite high risk behavior as compared to a seroconverter cohort [25]. Furthermore, no effect of the 1650G allele on the HIV-1 susceptibility and replication was observed in an in vitro replication assay using PHA stimulated PBMC from donors who were homozygous for the 1650A allele or heterozygous for the 1650G allele. However, in vitro infection of PHA stimulated PBMC may be not reflective of in vivo transmission conditions.

The observed differences between our present study and earlier reports [25], [26] may be explained by differences in the study populations. Interactions with other sequence variations that are distributed differently between the populations, immunological differences, different HIV-1 transmission routes, and differences in the circulating virus populations may account for the variable outcomes of these studies. Variations in natural history of HIV-1 infection among different HIV-1 infected cohorts have been reported before. For example, heterozygosity for the 32 basepair deletion in the CCR5 gene has a strong protective effect in the cohort of HIV-1 infected MSM of the ACS [18] whereas no effect on disease progression was observed in HIV-1 infected DU of the ACS [29].

Polymorphisms in the promoter region of CypA may have an effect on the CyPA expression levels and recently it was demonstrated that the 1604G allele enhances binding of transcription factors such as SP1, resulting in increased transcription of the CypA gene [25], [30]. Seemingly in contrast, we here observed a reduction in CypA expression in PBMC isolated from healthy C1604G heterozygous blood donors when compared to PBMC from carriers of the major allele. However, another polymorphism in the regulatory region of CypA, (C1575G), which is in 100% linkage disequilibrium with the C1604G polymorphism, was shown to reduce nuclear factor binding and decrease transcription of CypA [25]. Our results may thus indicate that the effect of these polymorphisms on CypA transcription is dominated by 1575G allele. However, the reduction in CypA expression levels had no effect on HIV-1 replication in vitro using PHA stimulated PBMC from donors who were homozygous or heterozygous for the C1604G genotype.

Overall, we here show that polymorphisms in the regulatory region of the CypA are only weakly associated with the clinical course of infection in the ACS. However, considering the important role of this protein in HIV-1 replication, manipulation of CypA activity by therapeutic intervention should be seriously considered.

## Materials and Methods

### Study participants

The study population, 364 Caucasian, MSM enrolled in the Amsterdam Cohort studies (ACS) on the natural history of HIV-1 infection between October 1984 and March 1986, was previously described [18]. The censor date of our study was set at the first day of effective antiretroviral therapy of the participant or end of follow-up. Of the 364 participants, 131 seroconverted during the study. The remaining 233 men were positive for HIV-1 antibodies at entry between October 1984 and April 1985. In previous epidemiological studies, the time since seroconversion of these prevalent cases has been estimated based on the incidence of HIV-1 infection amongst MSM of the Amsterdam Cohort and was on average 1.5 years before entry into the cohort studies [31]. For analysis, we combined the 131 participants with documented seroconversion and 233 seroprevalent participants with an imputed seroconversion date as one study group, since previous studies have not revealed differences in AIDS-free survival between the two groups [21]. From 334 participants DNA samples were available for analysis.

Sixty-eight MSM with high-risk behavior that persistently remained HIV-seronegative (HRSN) were retrospectively selected from the ACS. They remained seronegative during a follow up of 5 years before January 1996 and had reported unprotected receptive anal intercourse with at least 2 different partners before January 1996 (introduction of highly active antiretroviral therapy (HAART)).

A second study population consists of 120 HIV-1 infected intravenous drug users (DU) that participate in the Amsterdam Cohort Studies on HIV and AIDS among intravenous drug users which started enrolment in 1985. Asymptomatic men and women who were living in the Amsterdam area and who reported intravenous drug use in the preceding 6 months were enrolled in a prospective study on the prevalence and incidence of HIV-1 infection and risk factors for AIDS. In our study, we included 84 individuals who seroconverted for HIV antibodies during active follow-up. The median time between the last negative test and the first positive test for this group of prospective seroconverters was 3.9 months (interquartile range, 3.7–5.1). At entry in the cohort study 37 individuals were positive for HIV antibodies but had a negative test result before entry (i.e., retrospective seroconverters). Negative test results were obtained from samples taken for hepatitis B virus serology. The median interval between the last negative and first positive tests was 4.1 years (interquartile range, 1.8–5.4) for these seroconverters. For all 120 HIV infected DU (76 men and 44 women) used for our present analysis, the imputed seroconversion date [31] was calculated and used for further analysis.

Healthy volunteer blood donors (n = 104) were used as HIV-1 negative control group. The ACS has been conducted in accordance with the ethical principles set out in the declaration of Helsinki and written informed consent is obtained prior to data collection. The study was approved by the Academic Medical Center institutional medical ethics committee.

### Genotyping for regulatory polymorphisms in CypA

DNA samples of 334 HIV+ MSM, 68 HRSN, 120 HIV+ DU and 104 healthy controls were available for genotyping. For analysis of the polymorphism in the promoter region of CypA, DNA samples were amplified by PCR using Taq DNA polymerase (Invitrogen) in the presence of 3 mM MgCl with primer pair C1604G-F (5′-GCACTGTCACTCTGGCGAAGTCGCAGAC-3′) and P4H-R (5′-GCCGAGCACGTGCGTCGGACAGGAC-3′). The following amplification cycles were used: 5 min 95°C; 40 cycles of 30 s 95°C, 45 s 65°C, 60 s 72°C; 10 min 72°C. Subsequently PCR products were subjected to a restriction digest with 1U BstN1 (1 hour 60°C; New England Biolabs) to detect the C1604G polymorphism or with 10U Rsa1 (1 hour 37°C; New England Biolabs) to detect the A1650G polymorphism and analyzed on a 1.25% agarose gel. A PCR product containing a C at position 1604 or a G at position 1650 will result in a 524 bp fragment (undigested) product. A PCR product containing a C at position 1604 will result in digestion of the PCR product by BstN1 into a 423 bp and 101 bp fragments. A PCR product containing an A at position 1650 will be digested with Rsa1 resulting in a 369 bp and 55 bp fragments.

### CypA expression analysis and in vitro replication assay

Cells from 28 healthy blood donors were isolated, genotyped for C1604G and A1650G and the CypA expression levels were measured by real time Light cycler PCR. RNA was isolated by Trizol (Invitrogen) according to the manufacturer's protocol. Subsequently, cDNA was prepared using the SuperScript™ First-Strand Synthesis System for RT-PCR (Invitrogen). CypA mRNA levels were analyzed by SYBR green qPCR using the LightCycler (Roche). The reaction mix contained 20 mM Tris-HCl (pH 8.4), 50 mM KCl, 3 mM MgCl_2_, 200 µM dNTP, 250 µg/ml BSA, 500 nM primers, SYBR green I nucleic acid gel stain 40.000× diluted in water, and 0.6 U platinum Taq DNA polymerase (Invitrogen). The following primer sets were used for detection of CypA cDNA: CypA-2F: 5′-CTTCATCCTAAAGCATACGG-3′ and CypA-2R: 5′-CTTCTTGCTGGTCTTGCC-3′. Serial dilutions of plasmid DNA containing cDNA of CypA were used as a standard curve. To correct for differences in the cDNA input, levels of β-actin cDNA were analyzed by a SYBR green qPCR using the following primer set: BA-RNA-F 5′-GGCCCAGTCCTCTCCCAAGTCCAC-3′ and BA-RNA-R 5′-GGTAAGCCCTGGCTGCCTCCACC-3′. A serial dilution of 8E5 cells was used as a standard curve for β-actin. SYBR green qPCR was performed using the following program on the LightCycler: (1) preincubation and denaturation: 50°C for 2 min, 95°C for 2 min; (2) amplification and quantification: 45 cycles of 95°C for 5 sec, 55°C for 15 sec, 72°C for 15 sec; (3) melting curve: 95°C for 0 sec, 65°C for 15 min, 95°C for 0 sec with a temperature transition rate of 0.1°C/sec. Specificity of the PCR products measured using the SYBR green method was confirmed by a melting curve.

The replicative capacity of HIV-1 in PBMC from healthy blood donors that were genotyped for C1604G and A1650G was investigated. PBMC were isolated from buffy coats obtained from healthy seronegative blood donors by Ficoll-isopaque density gradient centrifugation. Cells (5×10^6^/ml) were stimulated for 2 days in Iscove's modified Dulbecco medium (Whitaker) supplemented with 10% fetal calf serum (Hyclone), penicillin (Gibco Brl) (100 U/ml), streptomycin (Gibco Brl) (100 µg/ml) and phytohemagglutinin (Welcome) (5 µg/ml). Cells were inoculated with 2000 TCID_50_ NL4-3(Ba-L) and replication was assessed by measuring virus production in culture supernatants by in-house p24 antigen capture enzyme-linked immunosorbent assay (ELISA) [32].

### Statistical analysis

Statistical analysis was performed employing SPSS (version 16). The genetic effects of SNPs on HIV-1 susceptibility were assessed by comparing the allelic and genotypic frequencies between susceptibility groups using the Pearson chi square test. The minor allele frequencies were compared to the most common genotype as a reference group. Kaplan Meier and Cox proportional hazard analysis were performed to study the genotypic association and relative hazard (RH) between the C1604G and A1650G polymorphisms in the regulatory regions of the CypA gene and disease progression. The following endpoints were considered for analysis: 1) AIDS according to the 1987 Centers for Disease Control and Prevention (CDC) definition [27]; 2) AIDS according to the 1993 CDC definition [28]; 3) CD4+ T cell counts below 200 cells/µl blood and 4. Viral RNA load above 10^4.5^ copies per ml plasma. CypA expression and differences in replicative capacity of HIV-1 in genotyped donor PBMC were compared using the student T-test. A result was considered significant if the P-value was <0.05.
